# The Association between Hyperhomocysteinemia and Thoracoabdominal Aortic Aneurysms in Chinese Population

**DOI:** 10.1155/2020/4691026

**Published:** 2020-07-28

**Authors:** Jianqing Deng, Jie Liu, Long Cao, Qun Wang, Hongpeng Zhang, Xiaoping Liu, Wei Guo

**Affiliations:** ^1^Department of Vascular and Endovascular Surgery, The First Medical Centre, Chinese PLA General Hospital, Beijing, China; ^2^Department of General Surgery, Chinese PLA No.983 Hospital, Tianjin, China; ^3^Department of Neurosurgery, The First Medical Centre, Chinese PLA General Hospital, Beijing, China

## Abstract

**Objective:**

To shed light on the association between hyperhomocysteinemia (HHcy) and thoracoabdominal aortic aneurysms (TAAAs).

**Methods:**

From July 2013 to March 2017, we conducted a matched case–control study involving individuals who presented to the Chinese People's Liberation Army General Hospital and underwent thoracoabdominal magnetic resonance angiography or computed tomography angiography. A total of 73 patients with TAAAs were enrolled in the case group, and 219 sex-matched subjects without TAAAs were included in the control group. We then examined the relationship between HHcy and TAAAs by logistic regression models and subgroup as well as interaction analyses.

**Results:**

Serum total homocysteine (tHcy) concentrations and the proportion of HHcy were significantly higher in the patients with TAAAs than in those without TAAAs (*P* < 0.001). Furthermore, the multivariate logistic regression models indicated that participants with HHcy had a 2.14-fold higher risk of TAAAs than those with a normal serum tHcy level (adjusted odds ratio (OR), 2.14; 95% confidence interval, 1.00–4.56). Similarly, each 1 *μ*mol/L increase in the serum tHcy concentration was associated with a 4% higher risk of TAAAs (adjusted OR, 1.04; 95% confidence interval, 1.00–1.07). Subgroup analyses indicated that HHcy tended to be associated with a greater risk of TAAAs in all stratified subgroups (adjusted ORs > 1). Furthermore, the interaction analyses revealed no interactive role in the association between HHcy and TAAAs.

**Conclusions:**

The present case–control study suggests that HHcy is an independent risk factor for TAAAs. Larger prospective cohort studies are warranted to validate these findings.

## 1. Introduction

A thoracoabdominal aortic aneurysm (TAAA) is defined as a permanent and continuous dilatation of the descending thoracic aorta and abdominal aorta [1]. Although TAAAs reportedly have a low incidence of 5.9 cases per 100,000 inhabitants annually [2], surgical repair of TAAAs is associated with a perioperative mortality rate ranging from 7.5% to 46.1% [3, 4]. In addition, one study showed that the 5- and 10-year survival rates of patients with TAAAs undergoing open repair were 63.6% and 36.8%, respectively [3], indicating that the prognosis of patients with TAAAs is far from satisfactory. Consequently, it is essential to decrease the incidence of TAAAs by discovering and controlling their risk factors.

Many patients with TAAAs are asymptomatic, and these TAAAs may remain undiagnosticated for years [5]. The risk of rupture increases with the aortic diameter, and the risk dramatically increases at diameters > 7 cm [1, 5]. Most ruptured TAAAs are catastrophic. Among patients with ruptured TAAAs who are able to undergo surgical repair, the operative mortality rate is reportedly as high as 46.1%, which is significantly higher than that of unruptured TAAA repair (15.9%) [4]. Therefore, the natural history of TAAAs causes many patients to easily miss the optimal timing of intervention. Identifying the biomarkers that indicate the presence of TAAAs might benefit early detection and intervention and thus improve the prognosis.

The interaction between media degeneration of the aortic wall and hemodynamic tension leads to the majority of TAAAs [1]. During the last few decades, hypertension, hyperlipidemia, obesity, smoking, a family history, and connective tissue diseases have been described as risk factors for TAAAs [1, 6], indicating that many risk factors for TAAAs are similar to those for atherosclerosis. Hyperhomocysteinemia (HHcy), defined as an elevated serum total homocysteine (tHcy) level, is associated with atherosclerotic diseases and intracranial aneurysms as well as abdominal aortic aneurysms (AAAs) [7–13]. However, no study has been performed to investigate the association between HHcy and TAAAs. Therefore, we performed a matched case–control study to explore the relationship between the serum tHcy level and TAAAs. We also performed subgroup and interaction analyses to detect the interactions of HHcy with other conventional factors in the association with TAAAs.

## 2. Patients and Methods

### 2.1. Study Design and Population

From July 2013 to March 2017, we conducted a matched case–control study involving individuals who presented to the Chinese People's Liberation Army General Hospital and underwent thoracoabdominal magnetic resonance angiography or computed tomography angiography. Consecutive patients who were newly diagnosed with TAAAs in the Department of Vascular and Endovascular Surgery were enrolled in the case group. These patients were matched 1 : 3 by sex with controls without TAAAs in the Health Management Center and were recruited during the same period (within 3 months). Patients with TAAAs caused by aortic dissection, trauma, or infection were excluded from the case group. The other exclusion criteria for all subjects were a history of vitamin B, folate, or antiepileptic medication use; a history of contraception and/or hormone replacement therapy; a chronic history of connective tissue disease (such as the Marfan syndrome, Loeys–Dietz syndrome, or Ehlers–Danlos syndrome), Takayasu arteritis, or aortitis; a history of serum tHcy-associated disease such as a malignant tumor or hypothyroidism; a history of mental disorders or diseases; and pregnancy. Our study was approved by the Ethics Committee of the Chinese People's Liberation Army General Hospital. We obtained a written informed consent from all participants, and all procedures in our study adhered to the principles of the Declaration of Helsinki.

Finally, 292 participants met the criteria (73 in the case group and 219 in the control group) during the study period and were recruited in our study.

### 2.2. Data Collection

All subjects underwent a medical consultation, standard physical examination. A uniform questionnaire was administered to record the participants' demographic characteristics and medical history, including age, sex, body mass index (BMI), smoking and drinking habit, hypertension, diabetes mellitus, hyperlipidemia, ischemic stroke, and coronary artery disease (CAD). The subjects filled out the questionnaire according to the guidance provided by trained doctors. A smoking habit was defined as consuming ≥100 cigarettes throughout life, and a drinking habit was defined as drinking ≥50 mL of alcohol per week for 6 months [8]. Hypertension was defined as receiving antihypertensive treatment or having a systolic blood pressure of ≥140 mmHg or diastolic blood pressure of ≥90 mmHg. Diabetes mellitus was defined as a glycated hemoglobin level of ≥6.5% or current treatment with medication for diabetes. Moreover, hyperlipidemia was defined as either the use of hypolipidemic agents or a serum total cholesterol concentration of >5.7 mmol/L, triglyceride concentration of >1.7 mmol/L, low-density lipoprotein cholesterol (LDL) concentration of >3.4 mmol/L, and high-density lipoprotein cholesterol (HDL) concentration of <1.0 mmol/L [8]. The medical histories of CAD and ischemic stroke were self-reported and confirmed by the subjects' families.

All subjects underwent blood biochemical tests for either a preoperative evaluation or health check-up. We retrieved the value of the serum tHcy, glucose, creatine, uric acid, total cholesterol, triglyceride, HDL, and LDL concentrations from the medical records. The blood biochemical test was performed as follows. After an 8-hour overnight fast, blood samples were withdrawn from the subjects and placed in a refrigerator at 4°C. The serum was centrifuged for separation within 1 hour and then stored at −80°C until analysis. All biochemical parameters were measured using a Roche Modular chemistry analyzer (Roche Diagnostics, Basel, Switzerland) at the clinical laboratory of our hospital. The intra- and interassay coefficients of variation were <5% for all of the assays performed. The estimated glomerular filtration rate (eGFR) was calculated by the Chronic Kidney Disease Epidemiology Collaboration equation based on the serum creatinine concentration [14].

### 2.3. Statistical Analysis

To minimize selection bias that might occur if we had excluded participants with missing data on BMI, LDL, HDL, glucose, uric acid, or creatine (<1%), we used multivariate imputation by chained equations to impute missing values. Five datasets were generated, and the results were pooled according to Rubin's rules. Categorical variables are summarized as frequency and percentage, and continuous variables are presented as mean and standard deviation. The Chi-squared test or Mann–Whitney test was used to compare the distributions of demographic and clinical characteristics between the TAAAs and control groups. Next, we performed a univariable logistic regression analysis to detect the relationship between TAAAs and conventional risk factors including age, sex, smoking and drinking habit, diabetes, hypertension, CAD, ischemic stroke, hyperlipidemia, eGFR, and BMI. Continuous parameters including age, eGFR, and BMI were transformed into categorical variables based on the recognized clinical cutoff points or laboratory reference ranges in the logistic regression models. HHcy was defined as a circulating tHcy level ≥ 15 *μ*mol/L [15]. Next, the independent association between the serum tHcy level (as either a categorical variable or a nontransformed continuous variable as well as a log(e)-transformed continuous variable because the distribution of the tHcy level was found to be skewed toward the left) and TAAAs was determined by two different multivariable logistic regression models. Adjusted odds ratios (ORs) with corresponding 95% confidence intervals (CIs) were estimated. The minimally logistic regression model was adjusted for sex and age. The fully adjusted model was adjusted for sex, age, smoking habit, hypertension, BMI, and eGFR (variable changing the coefficient of the continuous tHcy level by ≥10% when adding it into the basic logistical model including only the serum tHcy level or when deleting it from the complete logistical model comprising all variables in the abovementioned univariable logistic regression analysis). Finally, interaction and subgroup analyses were performed, including sex, age, smoking habit, hypertension, BMI, and eGFR.

All statistical analyses were performed with the software packages R (http://www.R-project.org, The R Foundation, Vienna, Austria) and EmpowerStats (http://www.empowerstats.com, X&Y Solutions, Inc., Boston, MA, USA). A two-tailed *P* value of < 0.05 was considered statistically significant.

## 3. Results

### 3.1. Participants' Baseline Characteristics

The subjects' baseline characteristics are summarized in Table 1. Generally, patients with TAAAs were slightly older (64.59 ± 12.73 years vs. 59.29 ± 7.92 years, *P* < 0.001), and there were higher proportions of smokers (57.53% vs. 19.18%, *P* < 0.001), drinkers (34.25% vs. 18.72%, *P* < 0.01), those with hypertension (68.49% vs. 32.42%, *P* < 0.001), and those with a chronic medical history of CAD(23.29% vs. 9.59%, *P* < 0.01) compared with individuals in the control group. The rates of patients with a chronic medical history of diabetes, hyperlipidemia, and ischemic stroke were not significantly different between the two groups. Additionally, a significantly lower BMI (24.22 ± 3.78 kg/m^2^ vs. 26.07 ± 3.33 kg/m^2^, *P* < 0.001), serum HDL concentration (1.12 ± 0.32 mmol/L vs. 1.21 ± 0.32 mmol/L, *P* < 0.05), and eGFR (97.40 ± 30.21 mL/min per 1.73 m^2^ vs. 112.05 ± 22.53 mL/min per 1.73 m^2^, *P* < 0.001) were observed in patients with TAAAs, whereas there were no statistically significant differences in the blood pressure, serum concentrations of cholesterol, triglycerides, LDL, glucose, or uric acid between the two groups.

### 3.2. HHcy and tHcy Levels

**Figure 1:**
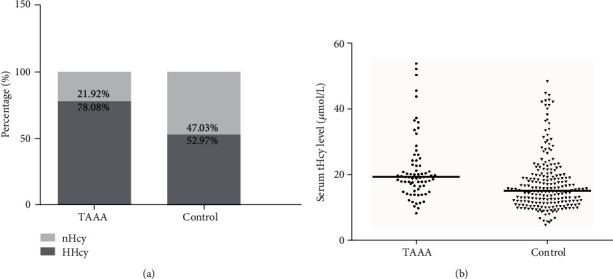
(a) Percentage of patients with HHcy in the two groups (nHcy: normal serum tHcy level; HHcy: hyperhomocysteinemia). (b) Distribution of serum tHcy levels in the two groups.

HHcy was found in 57 of 73 patients with TAAAs (78.08%) and 116 of 219 participants without TAAAs (52.97%), as is shown in Figure 1(a). The proportion of HHcy was significantly greater in the case than the control group (*P* < 0.001). Likewise, the serum tHcy level was significantly higher in the patients with TAAAs (median, 19.40; interquartile range, 16.50–23.10) than in those without TAAAs (median, 15.20; interquartile range, 11.60–19.90) with a *P* value of < 0.001 (Figure 1(b)).

### 3.3. Relationship between tHcy Level and TAAAs

The univariate logistic regression analyses indicated that age, hypertension, CAD, smoking and drinking habit, BMI, and eGFR were significantly associated with the presence of TAAAs (Table 2). The results of the multivariate logistic regression models are shown in Table 3. After adjustment for confounders, the serum tHcy level was independently associated with the risk of TAAAs in different multivariate logistic regression models (as either a categorical variable or continuous variable). Subjects with HHcy had a 2.14-fold higher risk of TAAAs than those with a normal serum tHcy level (adjusted OR, 2.14; 95% CI, 1.00–4.56). Similarly, each 1 *μ*mol/L increase in the serum tHcy concentration was associated with a 4% higher risk of TAAAs (adjusted OR, 1.04; 95% CI, 1.00–1.07). The relationship remained significant when the log(e)-transformed tHcy level was used in the multivariate logistic regression model (adjusted OR, 2.98; 95% CI, 1.36–6.53).

The results of the stratified and interaction analyses are presented in Table 4. The stratified analysis indicated no statistically significant association between HHcy and TAAAs in all stratified subgroups. Nevertheless, HHcy tended to be associated with a greater risk of TAAAs in all stratified subgroups (adjusted ORs > 1). Furthermore, the interaction analysis revealed no interactive role in the association between HHcy and TAAAs.

## 4. Discussion

### 4.1. Brief Discussion of Main Results and Implications

This paper describes the first case–control study to address the association between the serum tHcy level and risk of TAAAs. Our main finding is that an elevated serum tHcy level is independently associated with a higher risk of TAAAs. This association was robust when the serum tHcy concentration was included as either a nontransformed or log(e)-transformed continuous variable or a categorical variable in the same multivariate logistic regression model and when different potential confounders were adjusted in two multivariate logistic regressions. The stratified analyses revealed that HHcy was associated with a greater risk of TAAAs in all stratified subgroups (adjusted ORs > 1), although not statistically significant, which may have been a result of the limited sample size after stratification. Furthermore, no interactive role was found in the association between HHcy and TAAAs. Our main finding might have certain implications in identifying individuals at higher risk of developing TAAAs and tailoring more aggressive prevention. In addition, our results may help to detect higher risk among patients who already have TAAAs to provide earlier diagnostic strategies and treatments that can improve their prognosis.

### 4.2. Mechanisms

Homocysteine is a thiol-containing nonessential amino acid derived from the metabolism of the essential amino acid methionine [13]. Homocysteine can be degraded via the remethylation pathway in which 5-methyltetrahydrofolate is the substrate, employing vitamin B_12_ and folate as cofactors. It can also be converted into cysteine via the transsulfuration pathway catalyzed by cystathionine *β* synthase and vitamin B_6_ [16]. Consequently, the homocysteine level is affected by the folate and vitamin status. Previous studies have proven that an elevated serum tHcy level is associated with atherosclerotic diseases and intracranial aneurysms as well as AAAs [7–10, 13, 17]. In the present study, we showed a similar association between the tHcy level and TAAAs. The rarity of TAAAs has made the investigation of the molecular mechanisms underlying the association between HHcy and TAAAs difficult. However, basic research exploring the mechanisms underlying the association between HHcy and AAAs (the more common and closely related aortic aneurysms) has been more comprehensively conducted. These studies have provided important clues for us to explain and understand the association mechanically. From a molecular perspective, TAAAs might be associated with the same pathological features as AAAs, including degradation of the extracellular matrix, accumulation of reactive oxygen species, dedifferentiation or apoptosis of smooth muscle cells, and activation of various inflammatory cells [5, 18, 19]. HHcy may also mediate the formation of TAAAs through some of these pathogenetic pathways. Previous *in vitro* studies have shown that HHcy can increase serine elastase synthesis in aortic smooth muscle cells by 5- to 6-fold. Elastase leads to fragmentation of elastin, which provides the aorta's elasticity [20]. Elevated levels of tHcy also reportedly increase the secretion of elastolytic metalloproteinase-2 and metalloproteinase-9 in human endothelial cells [21]. These phenomena account for, at least in part, the degeneration of extracellular matrix in TAAAs. Previous studies have also shown that HHcy mediates formation of AAAs through recruitment of monocytes and macrophages to the aortic wall and activation or polarization of macrophages into inflammatory M1 cells [18, 22, 23], with subsequently aggravation of the aortic inflammation. Furthermore, homocysteine also directly interacts and activates the angiotensin II type I receptor to aggravate the vascular injury and then the development of AAAs [24]. It is reasonable to consider that the abovementioned mechanisms are also implicated in the association between HHcy and TAAAs. However, the pathological features of TAAAs and AAAs might be different to a certain extent [1]. Basic researches should be carried out to identify the specific mechanisms underlying the association between HHcy and TAAAs.

Additionally, longstanding atherosclerosis is thought to be the most frequent cause of degenerative aneurysms [6]. HHcy promotes atherosclerosis by causing vascular injury and adversely affecting several cellular functions, including lipid dysregulation, programmed cell death, and inflammation [25]. HHcy might also lead to TAAAs by aggravating atherosclerosis.

It might be effective to lower the serum tHcy level by folic acid and vitamin B_12_ or B_6_ supplementation to decrease the risk of TAAA formation in patients with HHcy. Future prospective studies are warranted to address this issue. However, a previous randomized controlled trial with a large population was performed to investigate the effect of tHcy-lowering therapy on the risk of major cardiovascular events in patients with vascular disease. The results showed that the tHcy-lowering treatments did not decrease the incidence of myocardial infarction, death of any cause, or adverse events compared with placebo. Only the incidence of stroke was reduced by tHcy-lowering interventions [26]. One possible reason for the failure to demonstrate certain clinical benefits of the tHcy-lowering interventions is that the study included patients who already had chronic tHcy elevation with vascular alterations that could not be reversed by vitamin supplementation [25]. Another reason may be that individuals with HHcy have an “Hcy memory effect” as a result of epigenetic alterations, which could continue to promote progression of cardiovascular complications even after the tHcy level is lowered [13]. Therefore, therapies targeting the epigenetic alterations should be developed to lower the risk of TAAA formation and atherosclerosis together with folic acid and vitamin B_12_ or B_6_ supplementation.

## 5. Limitations

This study has several limitations. First, our study was retrospective in nature, and recall bias cannot be totally avoided. For example, the participants' self-reported histories of chronic disease might have affected the accuracy of estimation of the disease prevalence. Second, the serum tHcy level was measured after the diagnosis of TAAAs; therefore, the causality of the association between an elevated tHcy level and TAAAs cannot be determined. Third, our study had a limited sample size, and only a few patients were included in each subgroup after stratifying by conventional risk factors, yielding limited statistical power. This limitation especially concealed some meaningful results in the stratified and interaction analyses. Fourth, this study mostly focused on the association between the tHcy level and TAAA occurrence, while further analysis involving the type, length, or maximum diameter of TAAAs was not performed. Fifth, the C677T polymorphism in methylenetetrahydrofolate reductase (MTHFR) closely affects the enzymatic activity of homocysteine metabolism. However, data on that genetic background was lacking, precluding assessment of the role of MTHFR C677T polymorphism in the association between serum tHcy concentration and TAAA formation. Finally, the serum levels of vitamin B_6_, vitamin B_12_, and folic acid were not measured, preventing us from assessing their interaction with the serum tHcy level.

Despite the aforementioned limitations, this is the first attempt to explore the association between the serum tHcy level and TAAAs and suggests that HHcy is independently associated with a higher risk of TAAA formation. We believe that our study can provide a foundation for subsequent larger prospective cohort studies.

## Figures and Tables

**Table 1:** Baseline characteristics of the study population.

Variables	All subjects	Control	TAAA	*P* value
*N*	292	219	73	
Age (years)	60.62 ± 9.61	59.29 ± 7.92	64.59 ± 12.73	<0.001
Sex				1.00
Female	36 (12.33%)	27 (12.33%)	9 (12.33%)	
Male	256 (87.67%)	192 (87.67%)	64 (87.67%)	
Smoking habits				<0.001
Yes	84 (28.77%)	42 (19.18%)	42 (57.53%)	
No	208 (71.23%)	177 (80.82%)	31 (42.47%)	
Drinking habits				0.006
Yes	66 (22.60%)	41 (18.72%)	25 (34.25%)	
No	226 (77.40%)	178 (81.28%)	48 (65.75%)	
Hypertension				<0.001
Yes	121 (41.44%)	71 (32.42%)	50 (68.49%)	
No	171 (58.56%)	148 (67.58%)	23 (31.51%)	
Diabetes				1.00
Yes	21 (7.19%)	16 (7.31%)	5 (6.85%)	
No	271 (92.81%)	203 (92.69%)	68 (93.15%)	
Hyperlipidemia				0.95
Yes	149 (51.03%)	112 (51.14%)	36 (49.32%)	
No	143 (48.97%)	107 (48.86%)	37 (50.68%)	
Ischemic stroke				0.098
Yes	19 (6.51%)	11 (5.02%)	8 (10.96%)	
No	273 (93.49%)	208 (94.98%)	65 (89.04%)	
CAD				0.004
Yes	38 (13.01%)	21 (9.59%)	17 (23.29%)	
No	254 (86.99%)	198 (90.41%)	56 (76.71%)	0.12
DBP (mmHg)	135.39 ± 17.90	134.15 ± 16.36	139.12 ± 21.57	
SBP (mmHg)	81.39 ± 9.58	81.42 ± 8.60	81.33 ± 12.11	0.58
Glucose (mmol/L)	5.18 ± 1.37	5.20 ± 1.47	5.11 ± 1.05	0.37
BMI (kg/m^2^)	25.61 ± 3.53	26.07 ± 3.33	24.22 ± 3.78	<0.001
eGFR (mL/min per 1.73 m^2^)	108.39 ± 25.45	112.05 ± 22.53	97.40 ± 30.21	<0.001
Urid acid (*u*mol/L)	338.81 ± 86.81	333.58 ± 81.67	354.49 ± 99.64	0.35
Cholesterol (mmol/L)	4.50 ± 0.94	4.52 ± 1.00	4.42 ± 0.75	0.87
Triglyceride (mmol/L)	1.45 ± 1.06	1.41 ± 0.94	1.56 ± 1.36	0.84
HDL (mmol/L)	1.19 ± 0.32	1.21 ± 0.32	1.12 ± 0.32	0.03
LDL (mmol/L)	2.79 ± 0.76	2.81 ± 0.80	2.74 ± 0.64	0.70

Abbreviations: TAAA: thoracoabdominal aortic aneurysm; CAD: coronary heart disease; DBP: diastolic blood pressure; SBP: systolic blood pressure; BMI: body mass index; eGFR: estimated glomerular filtration rate; HDL: high-density lipoprotein cholesterol; LDL: low-density lipoprotein cholesterol. For the continuous variables, the *P* value was calculated using the Mann–Whitney test. For the count variable, if there is a theoretical number < 10, the *P* value was calculated using the Fisher exact probability test. Otherwise, the Chi-squared test was applied.

**Table 2:** Univariable logistic regression models evaluating the association between the variables and TAAA.

Variables	Statistics	OR (95% CI)	P value
Sex			1.00
Female	36 (12.33%)	Ref.	
Male	256 (87.67%)	1.00 (0.45, 2.24)	
Age, years			<0.001
<60	143 (48.97%)	Ref.	
≥60	149 (51.03%)	2.64 (1.50, 4.62)	
Hypertension			<0.001
No	171 (58.56%)	Ref.	
Yes	121 (41.44%)	4.53 (2.56, 8.01)	
Diabetes			0.90
No	271 (92.81%)	Ref.	
Yes	21 (7.19%)	0.93 (0.33, 2.64)	
CAD			<0.01
No	254 (86.99%)	Ref.	
Yes	38 (13.01%)	2.86 (1.41, 5.79)	
Ischemic stroke			0.10
No	273 (93.49%)	Ref.	
Yes	19 (6.51%)	2.17 (0.86, 5.46)	
Smoking habits			<0.001
No	208 (71.23%)	Ref.	
Yes	84 (28.77%)	5.71 (3.22, 10.13)	
Drinking habits			<0.01
No	226 (77.40%)	Ref.	
Yes	66 (22.60%)	2.26 (1.25, 4.08)	
Dyslipidemia			0.95
No	143 (48.97%)	Ref.	
Yes	149 (51.03%)	0.98 (0.58, 1.67)	
BMI (kg/m^2^)			<0.01
<24	86 (29.45%)	Ref.	
≥24	206 (70.55%)	0.42 (0.24, 0.73)	
eGFR (mL/min per 1.73 m^2^)			<0.001
<90	39 (13.36%)	Ref.	
≥90	253 (86.64%)	0.11 (0.05, 0.24)	

Abbreviations: TAAA: thoracoabdominal aortic aneurysm; CAD: coronary heart disease; BMI: body mass index; eGFR: estimated glomerular filtration rate; Ref: reference.

**Table 3:** Logistic regression models evaluating the association between tHcy and TAAA.

Variable	No. (%) of participants		OR (95% CI)		
	Control	TAAA	Crude^a^	Minimally adjusted model^b^	Fully-adjusted model^c^
tHcy (*μ*mol/L)	219	73	1.04 (1.02, 1.07)	1.05 (1.02, 1.08)	1.04 (1.00, 1.07)
In (tHcy)			3.47 (1.85, 6.49)	3.75 (1.90, 7.39)	2.98 (1.36, 6.53)
tHcy level (*μ*mol/L)					
<15	103 (47.03%)	16(21.92%)	Ref.	Ref.	Ref.
≥15	116 (52.97%)	57(78.08%)	3.16 (1.71, 5.85)	3.10 (1.60, 6.01)	2.14 (1.00, 4.56)

Values in the table are OR (95% CI). Abbreviations: TAAA: thoracoabdominal aortic aneurysm; Ref: reference. ^a^Crude model adjust for: none. ^b^Minimally adjust model: adjust for sex, age (years). ^c^Fully adjust model: adjusted for sex, age (years), smoking, hypertension, BMI, and Cr-based eGFR (variables changing the coefficient of categorical tHcy level by >10%).

**Table 4:** The subgroup analyses and interaction analyses of association between HHcy and the risk of TAAA.

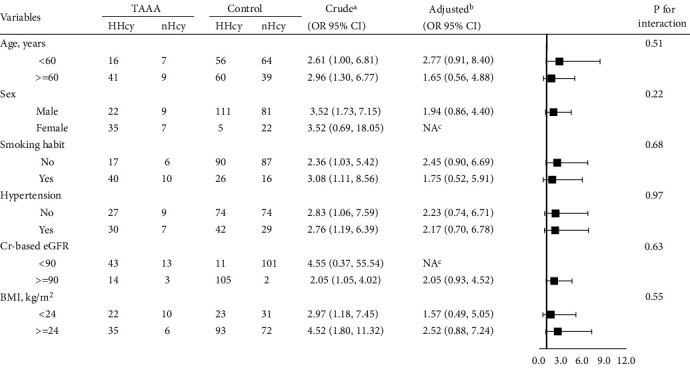

Abbreviations: TAAA: thoracoabdominal aortic aneurysm; BMI: body mass index; eGFR: estimated glomerular filtration rate. In each item, the model is not adjusted for the stratification variable. ^a^Crude model adjusted for none variable. ^b^Adjusted model adjusted for: sex, age (years), smoking, hypertension, categorical BMI, and categorical Cr-based eGFR. ^c^The model failed because of the small sample size.

## Data Availability

All of the data supporting the findings in this study are available from the corresponding author upon request.

## Abbreviations

HHcy: Hyperhomocysteinaemia
TAAAs: Thoracoabdominal aortic aneurysms
CAD: Coronary artery disease
AAAs: Abdominal aortic aneurysms
HDL: High-density lipoprotein cholesterol
LDL: Low-density lipoprotein cholesterol
eGFR: Estimated glomerular filtration rate
BMI: Body mass index.
